# Herbal Cannabis Use Is Not Associated with Changes in Levels of Endocannabinoids and Metabolic Profile Alterations among Older Adults

**DOI:** 10.3390/life12101539

**Published:** 2022-10-03

**Authors:** Ran Abuhasira, Shahar Azar, Alina Nemirovski, Joseph Tam, Victor Novack

**Affiliations:** 1 Clinical Research Center, Soroka University Medical Center and Faculty of Health Sciences, Ben-Gurion University of the Negev, Beer Sheva 8400101, Israel; 2 Obesity and Metabolism Laboratory, Institute for Drug Research, School of Pharmacy, Faculty of Medicine, The Hebrew University of Jerusalem, Jerusalem 9190401, Israel

**Keywords:** cannabis, marijuana, endocannabinoids, blood pressure, cardiovascular, older adults

## Abstract

**Simple Summary:**

The endocannabinoid system is a complex cell-signaling system that has numerous effects on the human body, including on the heart, blood vessels, and metabolism. In this study, we aimed to assess the effects of exogenous herbal medical cannabis use on the components of the endocannabinoid system among older adults with a diagnosis of hypertension. Medical cannabis is a product containing cannabinoids used for medical purposes. Herbal cannabis contains many types of cannabinoids, the most well-known of which are Δ^9^-tetrahydrocannabinol and cannabidiol. We followed people aged 60 years and older and conducted a number of tests, including endocannabinoids levels, before they started using cannabis and following three months of daily cannabis treatment. Fifteen patients (53.3% male; mean age, 69.5 years) underwent complete evaluations. We found positive correlations between the components of the endocannabinoid system and blood lipids, markers of inflammation, and blood pressure. On average, cannabis treatment for 3 months does not result in a significant change in the levels of endogenous cannabinoids and thus has a safe metabolic risk profile. This study provides additional evidence for the safety of medical cannabis use among older adults.

**Abstract:**

Activation of the endocannabinoid system has various cardiovascular and metabolic expressions, including increased lipogenesis, decreased blood pressure, increased heart rate, and changes in cholesterol levels. There is a scarcity of data on the metabolic effects of exogenous cannabis in older adults; therefore, we aimed to assess the effect of exogenous cannabis on endocannabinoid levels and the association with changes in 24 h ambulatory blood pressure and lipid levels. We conducted a prospective study of patients aged 60 years or more with hypertension treated with a new prescription of herbal cannabis. We assessed changes in endocannabinoids, blood pressure, and metabolic parameters prior to and following three months of cannabis use. Fifteen patients with a mean age of 69.47 ± 5.83 years (53.3% male) underwent complete evaluations. Changes in 2-arachidonoylglycerol, an endocannabinoid, were significantly positively correlated with changes in triglycerides. Changes in arachidonic acid levels were significantly positively correlated with changes in C-reactive protein and with changes in mean diastolic blood pressure. Exogenous consumption of cannabidiol was negatively correlated with endogenous levels of palmitoylethanolamide and oleoylethanolamide. On average, cannabis treatment for 3 months does not result in a significant change in the levels of endogenous cannabinoids and thus has a safe metabolic risk profile.

## 1. Introduction

Hypertension and dyslipidemia are common diseases, with an estimated prevalence of 45.6% and 38.1%, respectively, in the United States, primarily affecting older adults [1]. Hypertension is a complex condition that evolves by the integrated action of numerous systems in the human body, whereas the endocannabinoid system (ECS) is one of the less researched systems. It is composed of two endogenous ligands, 2-arachidonoylglycerol (2-AG) and *N*-arachidonoylethanolamide (anandamide, AEA), which are derivatives of arachidonic acid (AA) and their receptors, cannabinoid-1 and -2 receptors (CB_1_ and CB_2_). In addition, endocannabinoid-related compounds, such as oleoylethanolamide (OEA) and palmitoylethanolamide (PEA), are synthesized and degraded through the same enzymatic steps of AEA and 2-AG but do not bind to CB_1_ and CB_2_ and often exhibit actions opposite to those of endocannabinoids [2]. Current evidence show that endocannabinoid receptors are spread throughout the human body, specifically in the central nervous system, the immune system, the gastrointestinal system, adipose tissue, and the cardiovascular system [3]. Activation of the ECS has various cardiovascular and metabolic expressions, including increased lipogenesis, decreased blood pressure, increased heart rate, and changes in cholesterol levels [2]. The use of peripherally restricted CB_1_ receptor antagonists or inverse agonists leads to a reduction in body weight, adiposity, insulin resistance, and dyslipidemia in obese animal models [4]. The ECS was also proposed as a treatment target for a variety of metabolic disorders, including type 2 diabetes mellitus [5]. Levels of 2-AG, AEA, OEA, and PEA are significantly higher in obese than in normal-weight subjects [6]. Fasting salivary AEA and OEA directly correlate with BMI, waist circumference, and fasting insulin levels [6].

The interactions between blood pressure and exogenous cannabis are the subject of current research. Several studies have used an oral compound containing pure Δ^9^-tetrahydrocannabinol (THC) in older adults and reported mixed results. Some studies demonstrated a small increment in systolic blood pressure, causing a decrease or no change in heart rate and diastolic blood pressure [7,8], whereas other studies reported insignificant changes [9]. A large longitudinal study that assessed chronic recreational cannabis users did not report any significant association with hypertension [10]. The use of cannabidiol (CBD) caused a reduction in resting blood pressure in young healthy volunteers, an effect that was lost after seven days of treatment (tolerance) [11,12]. In chronic cannabis users, abrupt cessation of cannabis led to an increase in blood pressure [13]. Another study that assessed young healthy chronic cannabis smokers showed that cannabis smoking was associated with visceral adiposity and adipose tissue insulin resistance but not with hepatic steatosis, insulin insensitivity, impaired pancreatic β-cell function, or glucose intolerance [14]. Despite these numerous known effects of the ECS, the effect of exogenous cannabis on the ECS and its constituents remains poorly understood. Assessment of plasma AEA and 2-AG levels immediately following cannabis smoking did not reveal any significant changes relative to baseline in chronic recreational cannabis users [15].

In recent years, the use of medical cannabis has rapidly increased [16,17,18], in addition to vast changes in regulations [19,20]. Thus, there is an increasing need to understand the relationship between the use of cannabis, the ECS, blood pressure, and lipid homeostasis. We aimed to assess the effect of exogenous cannabis administration in older adults with hypertension on endocannabinoid levels and the association with changes in 24 h ambulatory blood pressure and lipids levels.

## 2. Materials and Methods

### 2.1. Study Design and Population

This is a multicenter, prospective, observational study conducted between November 2018 and May 2020 at three sites in Israel. Participants were recruited from outpatient clinics at three sites. Inclusion criteria were 60 years of age or older, prior diagnosis of primary hypertension, and a recommendation to prescribe medical cannabis from the treating physician. Further study criteria were previously published [21].

### 2.2. Procedures

Baseline evaluations included blood pressure assessment via 24 h ambulatory blood pressure measurement (ABPM), electrocardiogram, anthropometric measurements, metabolic blood tests, demographic data, and general medical history. The composition of the cannabis, its method of administration, and dosing were all decided by the treating physician. Following three months of daily cannabis use, we evaluated all patients by conducting the same assessments as in the first visit.

### 2.3. Assessments and Outcomes

Metabolic assessment through blood tests included serum cholesterol, triglycerides, hemoglobin A1c (HbA1c), fasting serum glucose, fasting insulin, C-reactive protein (CRP), kidney function tests, and electrolytes. After blood was collected from the patients, the samples were kept for 30 min at room temperature and centrifuged at 2000 rpm, and after several minute, the serum was placed in a −80 °C freezer until further analysis of endocannabinoids.

### 2.4. Endocannabinoid Measurements

**Figure 1:**
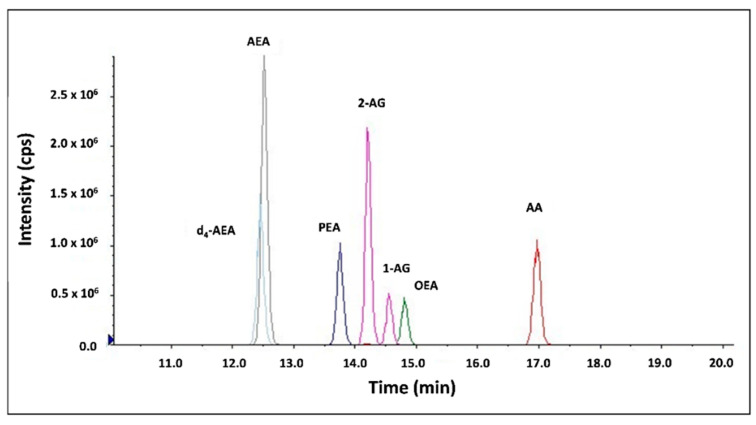
LC-MS/MS chromatogram.

AEA, 2-AG, AA, OEA, and PEA were extracted, purified, and quantified in serum by stable isotope dilution liquid chromatography/tandem mass spectrometry (LC-MS/MS) as previously described [22,23,24]. LC-MS/MS analyses were conducted on a Sciex (Framingham, MA, USA) QTRAP^®^ 6500+ mass spectrometer coupled with a Shimadzu (Kyoto, Japan) UHPLC system. Liquid chromatographic separation was obtained using 5 μL injections of samples onto a Kinetex 2.6 μm C18 (100 × 2.1 mm) column from Phenomenex (Torrance, CA, USA). The autosampler was set at 4 °C, and the column was maintained at 40 °C during the entire analysis. Endocannabinoids were detected in positive ion mode using electron spray ionization (ESI) and a multiple reaction monitoring (MRM) mode of acquisition, using d_4_-AEA as internal standard (IS). The IonDrive^TM^ Turbo V source temperature was set at 450 °C with an ion spray voltage of 4000 V. The curtain gas was set at 30.0 psi. The nebulizer gas (Gas 1) was set to 40 psi, and the turbo heater gas (Gas 2) was set to 40 psi. The dwell time was set to 30 ms. The collision energy (CE), declustering potential (DP), and collision cell exit potential (CXP) for the monitored transitions are listed in Table 1. The LC-MS/MS chromatogram is presented in Figure 1. The serum levels of AEA, 2-AG, OEA, PEA, and AA were measured in duplicate against standard curves.

### 2.5. Statistical Analysis

All statistical analyses are of paired data; therefore, we used McNemar’s test for categorical variables and a Wilcoxon test for continuous variables that were not normally distributed. We measured the association (both strength and direction) between endocannabinoids and the various blood test values using Kendall’s rank correlation coefficient (Kendall’s tau). We used SPSS (IBM SPSS version 25, Armonk, NY, USA) for statistical analysis.

## 3. Results

### 3.1. Cohort Characteristics

A total of 38 patients were recruited to the study, 26 of whom completed three months of cannabis treatment with a follow-up evaluation [21]. Of those, 15 participants had available endocannabinoids results. The median age of the patients was 69 years (IQR: 66–72), and 53.3% were male (Table 2). The most common indication for cannabis treatment was Parkinson’s disease (PD)-associated pain (46.7%), and most (80.0%) patients used cannabis oil orally, with the rest utilizing an inhalation method. The median dose of THC per day was 22.2 mg, and the median dose of CBD was 30.0 mg. Six (40%) patients used cannabis twice a day, five (33.3%) used cannabis once a day, and the rest used cannabis three times a day or more.

### 3.2. Blood Tests and Metabolic Parameters

**Figure 2:**
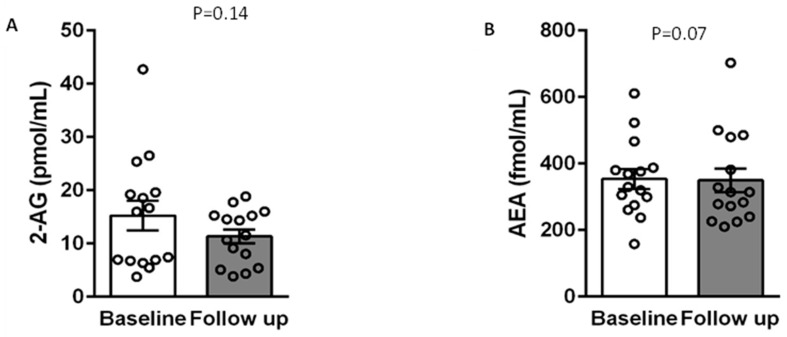

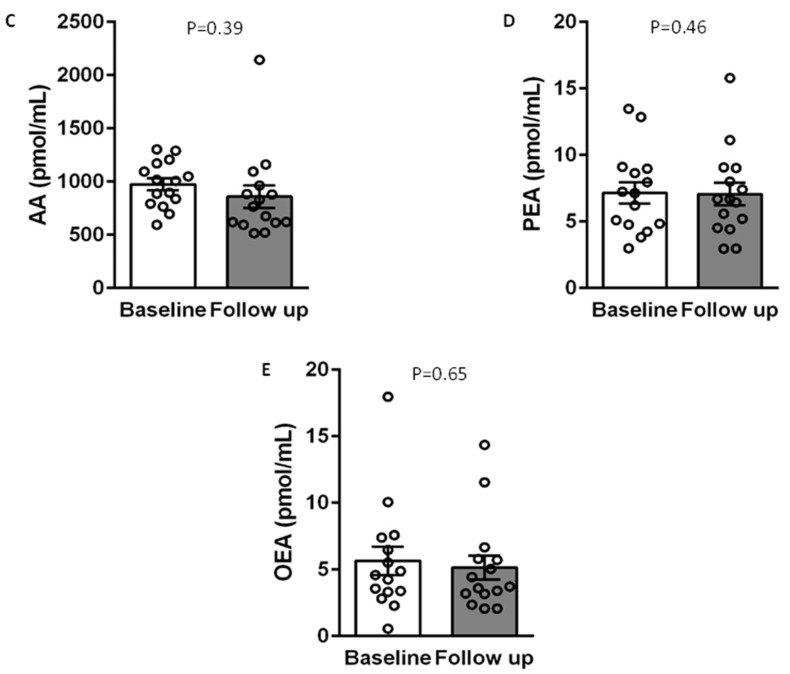
The distribution of 2-AG (**A**), AEA (**B**), AA (**C**), PEA (**D**), and OEA (**E**) among participants before and after 3 months of cannabis treatment. Each dot represents a patient, and the rectangles are the mean values of the entire group (*N* = 15). AEA—*N*-arachidonoylethanolamide (anandamide); 2-AG—2-arachidonoylglycerol; AA—arachidonic acid; OEA—oleoylethanolamide; PEA—palmitoylethanolamide.

On average, there were no significant changes in the investigated metabolic parameters or between levels of endocannabinoids prior to and following cannabis treatment. However, the mean values of all endocannabinoids were lower after cannabis treatment. Assessment of these parameters in each patient showed some variability in terms of the effect of cannabis among patients (Table 2 and Figure 2).

### 3.3. Correlations between Endocannabinoids, Metabolic Parameters, and Exogenic Cannabis

Changes in AA levels were significantly positively correlated with changes in CRP and with changes in mean diastolic blood pressure (τ = 0.39, *p* = 0.04 and τ = 0.45, and *p* = 0.03, respectively). Changes in 2-AG levels were significantly positively correlated with changes in triglycerides (τ = 0.47, *p* = 0.02). Total daily dose of cannabidiol (CBD) was negatively correlated with changes in PEA and OEA levels (τ = −0.44, *p* = 0.02 and τ = −0.40, *p* = 0.04, respectively), but there was no correlation observed between the total daily dose of THC and the levels of endocannabinoids in the blood (Table 3).

Patients with PD had higher baseline levels of 2-AG compared to patients with other indications for cannabis treatment: 19.2 ± 13.5 vs. 11.8 ± 6.7 pmol/mL (*p* = 0.1), respectively. However, 3 months after initiation of treatment, the levels were similar between the two groups: 11.6 ± 5.5 for the patients with PD compared to 11.1 ± 5.1 for the others (*p* = 0.9).

## 4. Discussion

In this prospective cohort study of older adults with hypertension, we found that treatment with cannabis for three months does not result in a significant change in the levels of endogenous cannabinoids and thus has a safe metabolic risk profile. The levels of 2-AG were positively correlated with plasma triglycerides, and exogenous consumption of CBD was negatively correlated with endogenous levels of PEA and OEA.

The positive correlation between 2-AG and plasma triglycerides is a well-established finding that has been reported in patients on maintenance hemodialysis, obese men, following bariatric surgery, and in patients with nonalcoholic fatty liver disease [24,25,26,27,28]. A previous study comparing patients with PD, either with or without levodopa-induced dyskinesias, to healthy individuals showed that patients with PD had significantly lower levels of 2-AG [29]. Our findings show that PD patients had higher levels of 2-AG before treatment, whereas after treatment, there was no difference between patients with and without PD. The cause of this phenomenon is currently unknown, but alterations in the ‘tone’ of the ECS in PD have been demonstrated in several studies [29,30].

A study on DOCA-salt rats (hypertension models) showed that exogenous CBD decreased the levels AEA, 2-AG, and OEA but did not modify blood pressure or heart rate [31]. A previous study assessed the immediate effect of exogenous THC on plasma levels of endocannabinoids, showing an increase followed by a decrease to a minimum level after 5 h in AEA and 2-AG following THC administration. The levels returned to baseline after 48 h [32]. Similar results were also reported 2–3 h after administration of THC capsules [33]. However, a study on heavy cannabis smokers did not show any difference in levels of AEA and 2-AG three hours after smoking a cannabis cigarette containing 5.6% THC [15]. Our findings with respect to administration of varying mixtures of CBD and THC showed a decrease in systolic and diastolic blood pressure and a positive correlation between the mean diastolic blood pressure values and AA levels. We did not observe any significant changes in AEA or 2-AG levels after treatment. All the abovementioned studies assessed the immediate short-term effect of exogenous cannabis, as opposed to the long-term effect, as assessed in our study. Studies assessing the long-term effects of exogenous cannabis on endocannabinoids are lacking [34].

A study that assessed overweight adults revealed that AEA, 2-AG, OEA, and PEA were all positively associated with body fat percentage and that OEA was positively associated with heart rate, total cholesterol, and LDL cholesterol, whereas PEA was positively associated with total cholesterol, LDL cholesterol, and HDL cholesterol [35]. Our findings are consistent with some of those results, as we found a positive correlation of PEA and OEA with HDL cholesterol but a negative correlation with LDL cholesterol, leading to a negative correlation with the LDL/HDL ratio. The net effect of exogenous cannabinoids on lipid metabolism is still being investigated [36].

In our study, some patients demonstrated substantial increases or decreases in endocannabinoid levels, whereas in others, the difference before and after the treatment was negligible. An attempt to analyze this heterogenous response and characterize the patients with the substantial differences did not yield any meaningful conclusions, probably due to the small sample size in each subgroup.

Our study is subject to some limitations. First, due to the lack of control group, the causality of cannabis effect cannot be inferred, and we can only determine association. However, every patient served as their own control, reducing the chance of showing effects that are completely spontaneous. Second, cannabis type, dosing, and route of administration were not identical among all patients. Third, the sample size used in this study is too small to conduct subgroup analyses and understand the characteristics of patients in which we might expect an increase or decrease in the levels of endocannabinoids.

## 5. Conclusions

Among older adults with hypertension, cannabis treatment for 3 months was not associated with a significant change in the levels of endocannabinoids and was associated with a safe metabolic risk profile. Levels of 2-AG were positively correlated with plasma triglycerides, and exogenous consumption of CBD was negatively correlated with endogenous levels of PEA and OEA. Further larger studies with appropriate controls are needed to explore the effects of exogenous cannabis on levels of endocannabinoids.

## Figures and Tables

**Table 1:** Optimized MRM transitions.

Compound	Molecular Ion [M+H]^+^ (m/z)	Fragment (m/z)	DP (volts)	CE (volts)	CXP (volts)
**2-AG**	379.2	287.1 (quantifier)	70	19	14
		91 (qualifier)	70	67	10
**AEA**	348.2	287.1 (quantifier)	26	13	16
		62 (qualifier)	26	13	8
**PEA**	300.3	283.2 (quantifier)	130	19	24
		62 (qualifier)	130	17	8
**AA**	305.3	91 (quantifier)	1	49	10
		287.1 (qualifier)	1	13	22
**OEA**	326.3	61.9 (quantifier)	146	21	24
		309.1 (qualifier)	146	21	42
**d_4_-AEA**	352.3	287.1 (quantifier)	66	15	20
		66 (qualifier)	66	21	8

AEA—*N*-arachidonoylethanolamide (anandamide); 2-AG—2-arachidonoylglycerol; AA—arachidonic acid; OEA—oleoylethanolamide; PEA—palmitoylethanolamide.

**Table 2:** Baseline characteristics of the patients.

Variable	Reference Range	Before Treatment	After Treatment	*p*-Value
Age (years, median, IQR)		69 (66–72)	–	–
Male (n, %)		8 (53.3%)	–	–
**Indication for cannabis (*n*, %)**			–	–
Neuropathic pain		5 (33.3%)	–	–
Parkinson’s Disease-associated pain		7 (46.7%)	–	–
Other indication		3 (20.0%)	–	–
**Mode of cannabis administration**			–	–
Oil		11 (73.3%)	–	–
Smoking		3 (20.0%)	–	–
Oil and smoking		1 (6.7%)	–	–
**CBD and THC composition of cannabis products at treatment initiation (n, %)**			–	–
THC 5%/CBD 10%		5 (23.8%)	–	–
THC 10%/CBD 2%		7 (33.3%)	–	–
THC 10%/CBD 10%		3 (14.3%)	–	–
THC 1%/CBD 20%		1 (4.8%)	–	–
THC 3%/CBD 15%		1 (4.8%)	–	–
THC 15%/CBD 3%		2 (9.5%)	–	–
THC 20%/CBD 1%		1 (4.8%)	–	–
**Cannabis dosing**		–	–	–
Cannabis administration once a day (n, %)		5 (33.3%)	–	–
Cannabis administration twice a day (n, %)		6 (40%)	–	–
Cannabis administration ≥3 times a day (n, %)		4 (26.7%)	–	–
Total THC dose per day (mg, median, IQR)		22.2 (13.5–40.0)	–	–
Total CBD dose per day (mg, median, IQR)		30.0 (5.5–39.5)	–	–
**Blood test values**			–	–
Urea (mg/dL, mean ± SD)	17–43	50.11 ± 21.7	51.19 ± 27.04	0.13
Uric acid (mg/dL, mean ± SD)	3.5–7.2 (male)	5.62 ± 1.41	5.54 ± 1.58	0.89
	2.6–6 (female)			
Creatinine (mg/dL, mean ± SD)	0.67–1.17 (male)	0.93 ± 0.27	0.88 ± 0.31	0.1
	0.51–0.95 (female)			
Sodium (mEq/L, mean ± SD)	135–145	139.93 ± 1.62	140 ± 1.85	0.84
Potassium (mEq/L, mean ± SD)	3.5–5.1	4.66 ± 0.43	5.05 ± 1.16	0.46
Chloride (mEq/L, mean ± SD)	98–106	103.87 ± 3.25	104 ± 3.09	0.34
Calcium (mg/dL, mean ± SD)	8.5–10.5	9.61 ± 0.34	9.37 ± 0.5	0.03
Total cholesterol (mg/dL, mean ± SD)		164.27 ± 57.7	164.07 ± 38.61	0.73
Non-HDL cholesterol (mg/dL, mean ± SD)		113.4 ± 53.94	112.33 ± 33.54	1
LDL cholesterol (mg/dL, mean ± SD)		78.86 ± 38.89	87.67 ± 31.93	0.57
HDL cholesterol (mg/dL, mean ± SD)		50.8 ± 12.39	51.93 ± 14.3	0.55
LDL/HDL Ratio		1.56 ± 0.66	1.75 ± 0.6	0.47
Triglycerides (mg/dL, mean ± SD)		137.13 ± 75.22	123.07 ± 42.87	0.46
Fasting plasma glucose (mg/dL, mean ± SD)	70–100	119.33 ± 30.01	112.93 ± 35.46	0.12
Hemoglobin A1C (%, mean ± SD)	4–5.7	6.15 ± 0.86	6.09 ± 0.88	0.86
Fasting insulin (mU/mL, mean ± SD)	5–25	14.32 ± 8.37	12.04 ± 8.53	0.21
HOMA-IR		4.55 ± 3.31	3.56 ± 2.9	0.1
C-reactive protein (mg/dL, mean ± SD)	0.02–0.5	0.65 ± 0.76	0.9 ± 1.31	0.89
**Endocannabinoids**				
2-AG (pmol/mL, mean ± SD)		15.23 ± 10.73	11.35 ± 5.1	0.07
AEA (fmol/mL, mean ± SD)		353.41 ± 114.5	349.57 ± 137.46	0.39
AA (pmol/mL, mean ± SD)		974.1 ± 216.13	859.27 ± 407.94	0.14
OEA (pmol/mL, mean ± SD)		5.65 ± 4.16	5.15 ± 3.5	0.65
PEA (pmol/mL, mean ± SD)		7.16 ± 3.11	7.06 ± 3.33	0.46

THC—tetrahydrocannabinol; CBD—cannabidiol; HOMA-IR—homeostasis model assessment of insulin resistance; LDL—low-density lipoprotein; HDL—high-density lipoprotein; AEA—*N*-arachidonoylethanolamide (anandamide); 2-AG—2-arachidonoylglycerol; AA—arachidonic acid; OEA—oleoylethanolamide; PEA—palmitoylethanolamide.

**Table 3:** Correlations between delta of changes relative to baseline and follow-up in endocannabinoids and metabolic parameters (τ, Kendall’s correlation).

Parameter	2-AG	AEA	AA	OEA	PEA
Mean diastolic blood pressure (mm Hg)	0.08	0.25	0.45 *	−0.12	−0.01
Mean heart rate (bpm)	−0.16	0.19	0.25	−0.32	−0.21
Mean systolic blood pressure (mm Hg)	0.01	0.10	0.25	−0.23	−0.08
Body mass index	−0.09	−0.18	−0.21	−0.30	0.00
Waist-to-hip ratio	0.23	−0.11	0.05	0.02	0.02
Total THC per day (mg)	−0.05	0.26	0.07	−0.01	−0.20
Total CBD per day (mg)	−0.04	0.04	−0.02	−0.40 *	−0.44 *
Total cholesterol (mg/dL)	−0.13	0.00	−0.06	−0.10	−0.21
LDL cholesterol (mg/dL)	−0.30	0.06	0.01	−0.28	−0.39
HDL cholesterol (mg/dL)	−0.20	0.18	0.16	0.24	0.09
Non-HDL Cholesterol (mg/dL)	−0.03	−0.05	0.05	−0.16	−0.24
LDL/HDL Ratio	−0.19	−0.05	−0.03	−0.47 *	−0.45 *
C-reactive protein (mg/dL)	−0.14	0.26	0.39 *	−0.01	−0.05
Triglycerides (mg/dL)	0.47 *	0.10	0.20	−0.01	0.03
Hemoglobin A1C (%)	0.16	0.36	0.20	0.13	0.18
Fasting plasma glucose (mg/dL)	0.12	0.04	−0.06	0.00	−0.10
HOMA-IR	0.14	−0.12	0.05	−0.01	−0.01

* *p*-value < 0.05, HOMA-IR—homeostasis model assessment of insulin resistance; LDL—low-density lipoprotein; HDL—high-density lipoprotein; AEA—*N*-arachidonoylethanolamide (anandamide); 2-AG—2-arachidonoylglycerol; AA—arachidonic acid; OEA—oleoylethanolamide; PEA—palmitoylethanolamide; THC—delta 9-tetrahydrocannabinol; CBD—cannabidiol.

## Data Availability

The data used in the analysis of this study are not publicly available due to national regulations but are available from the corresponding author upon request.
